# Supplementary material for: Functional morphology of a lobopod: case study of an onychophoran leg
Source: R Soc Open Sci. 2019 Oct 16;6(10):191200. doi: 10.1098/rsos.191200 (PMC6837196; doi:10.1098/rsos.191200)
Supplement: Figure S1 [file rsos191200supp1.pdf]

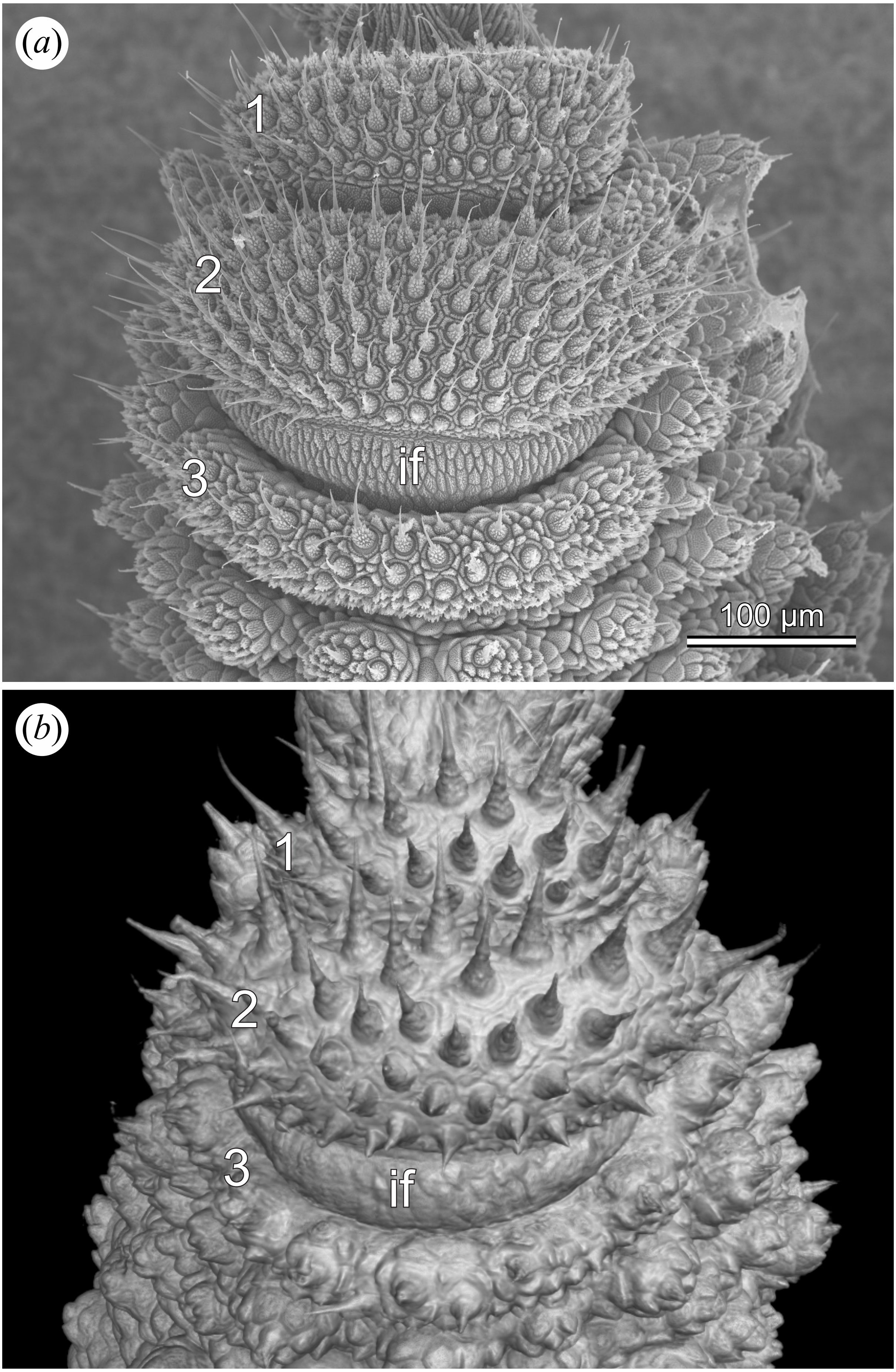

Supplementary Figure 1. Spinous pads of the lobopod in *E. rowelli*. Scanning electron micrograph (**a**) and volume rendering based on nanoCT data (**b**) from left mid-trunk leg in ventrodistal view. Distal is up in both images. Spinous pads are numbered. Note the position of the spineless integumentary fold between the second and third spinous pads. Abbreviation: if, spineless integumentary fold.
