# Supplementary material for: Functional morphology of a lobopod: case study of an onychophoran leg
Source: R Soc Open Sci. 2019 Oct 16;6(10):191200. doi: 10.1098/rsos.191200 (PMC6837196; doi:10.1098/rsos.191200)
Supplement: Figure S2 [file rsos191200supp2.pdf]

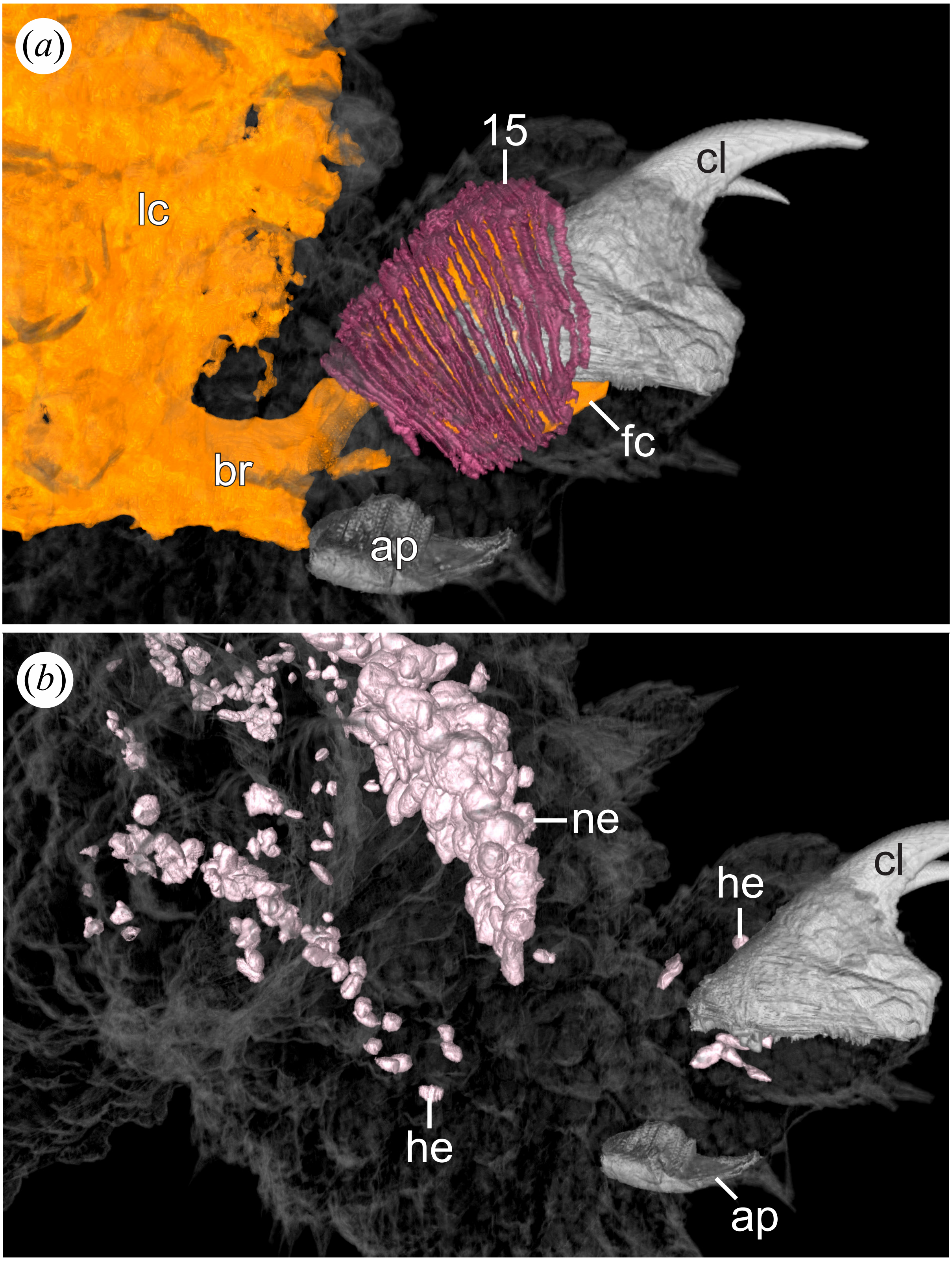

Supplementary Figure 2. Musculature and haemolymph system of the foot in *E. rowelli*. Volume rendering based on nanoCT data from left mid-trunk leg. Dorsal is up in both images. Body surface is semi-transparent. (**a**) Detail of the haemolymph channel within the foot bridge connecting the leg to the foot. The foot constrictor (#15, as in the main text) may regulate the hydrostatic pressure inside the foot. (**b**) Distribution of haemo- and nephrocytes within the leg and foot. Note that nephrocytes are larger and restricted to the leg cavity. Abbreviations: ap, foot apodeme; br, foot bridge; cl, claw; fc, foot cavity; he, haemocyte; lc, leg cavity; ne, nephrocyte.
