# Supplementary material for: Functional morphology of a lobopod: case study of an onychophoran leg
Source: R Soc Open Sci. 2019 Oct 16;6(10):191200. doi: 10.1098/rsos.191200 (PMC6837196; doi:10.1098/rsos.191200)
Supplement: Figure S3 [file rsos191200supp3.pdf]

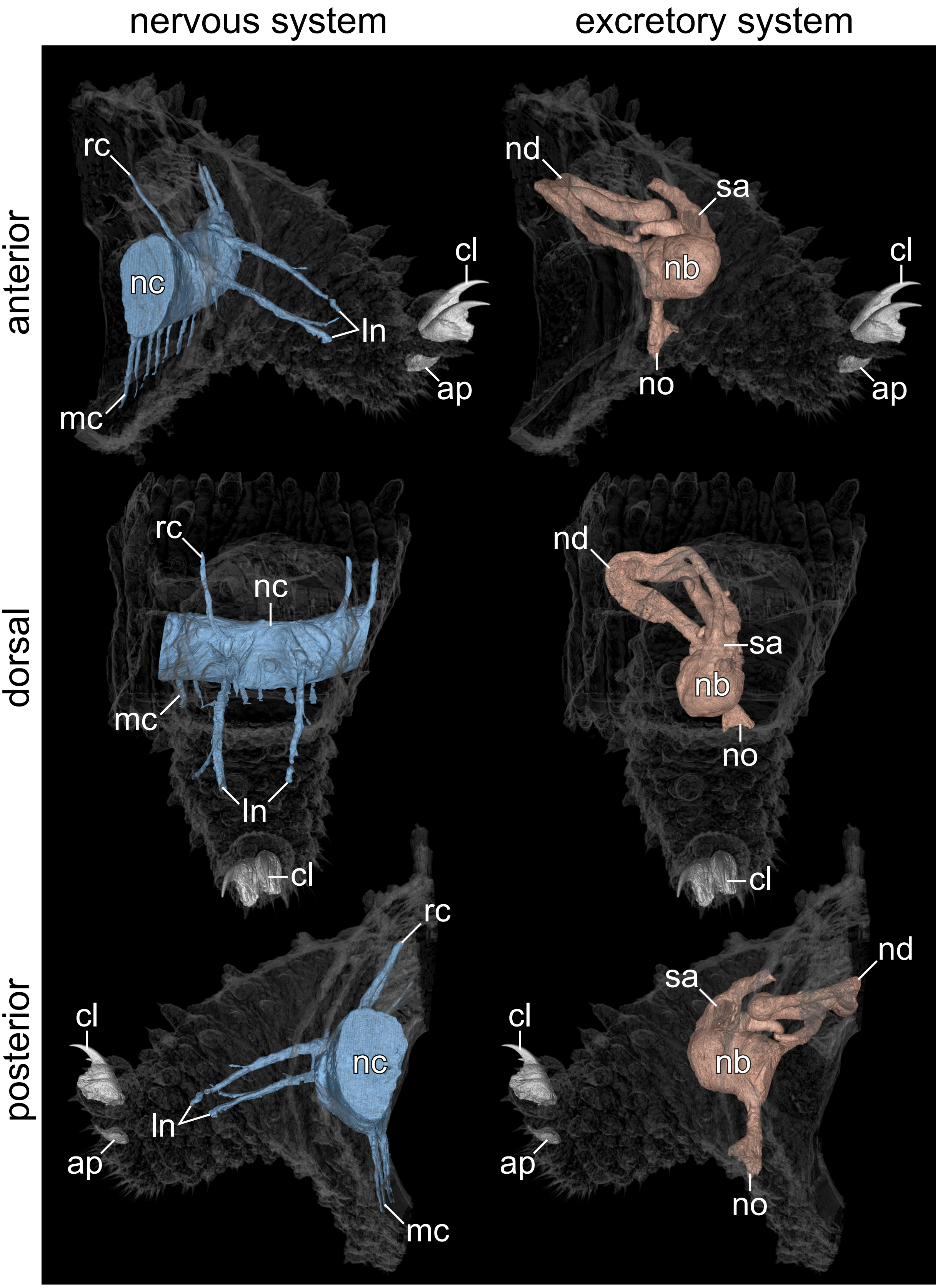

Supplementary Figure 3. Elements of the nervous and excretory system in the lobopod of *E. rowelli*. Volume rendering based on nanoCT data from left mid-trunk leg. Dorsal is up in all images. Body surface is semi-transparent. Abbreviations: ap, foot apodeme; cl, claw; ln, leg nerves; mc, median commissure; nb, nephridial bladder; nc, nerve cord; nd, nephridial duct; no, nephridial opening; rc, ring commissure; sa, sacculus.
