# Supplementary material for: Functional morphology of a lobopod: case study of an onychophoran leg
Source: R Soc Open Sci. 2019 Oct 16;6(10):191200. doi: 10.1098/rsos.191200 (PMC6837196; doi:10.1098/rsos.191200)
Supplement: Figure S4 [file rsos191200supp4.pdf]

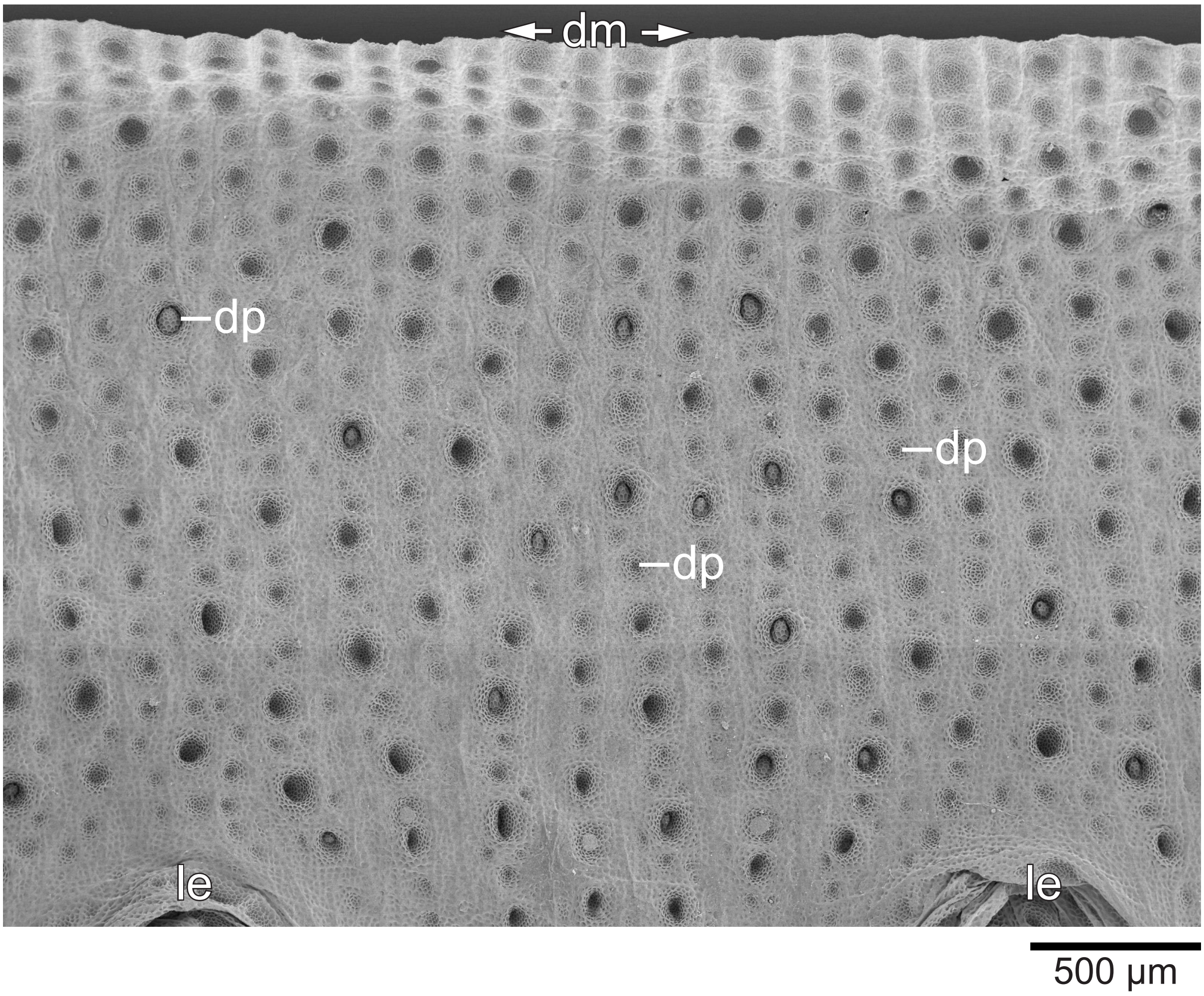

Supplementary Figure 4. Internal view of a moulted skin of *E. rowelli*. Scanning electron micrograph. Dorsal is up. Note the lack of apodeme-like structures on the dorsolateral body wall. Abbreviations: dm, dorsomedian furrow; dp, dermal papilla; le, leg.
