# Supplementary material for: Functional morphology of a lobopod: case study of an onychophoran leg
Source: R Soc Open Sci. 2019 Oct 16;6(10):191200. doi: 10.1098/rsos.191200 (PMC6837196; doi:10.1098/rsos.191200)
Supplement: Figure S5 [file rsos191200supp5.pdf]

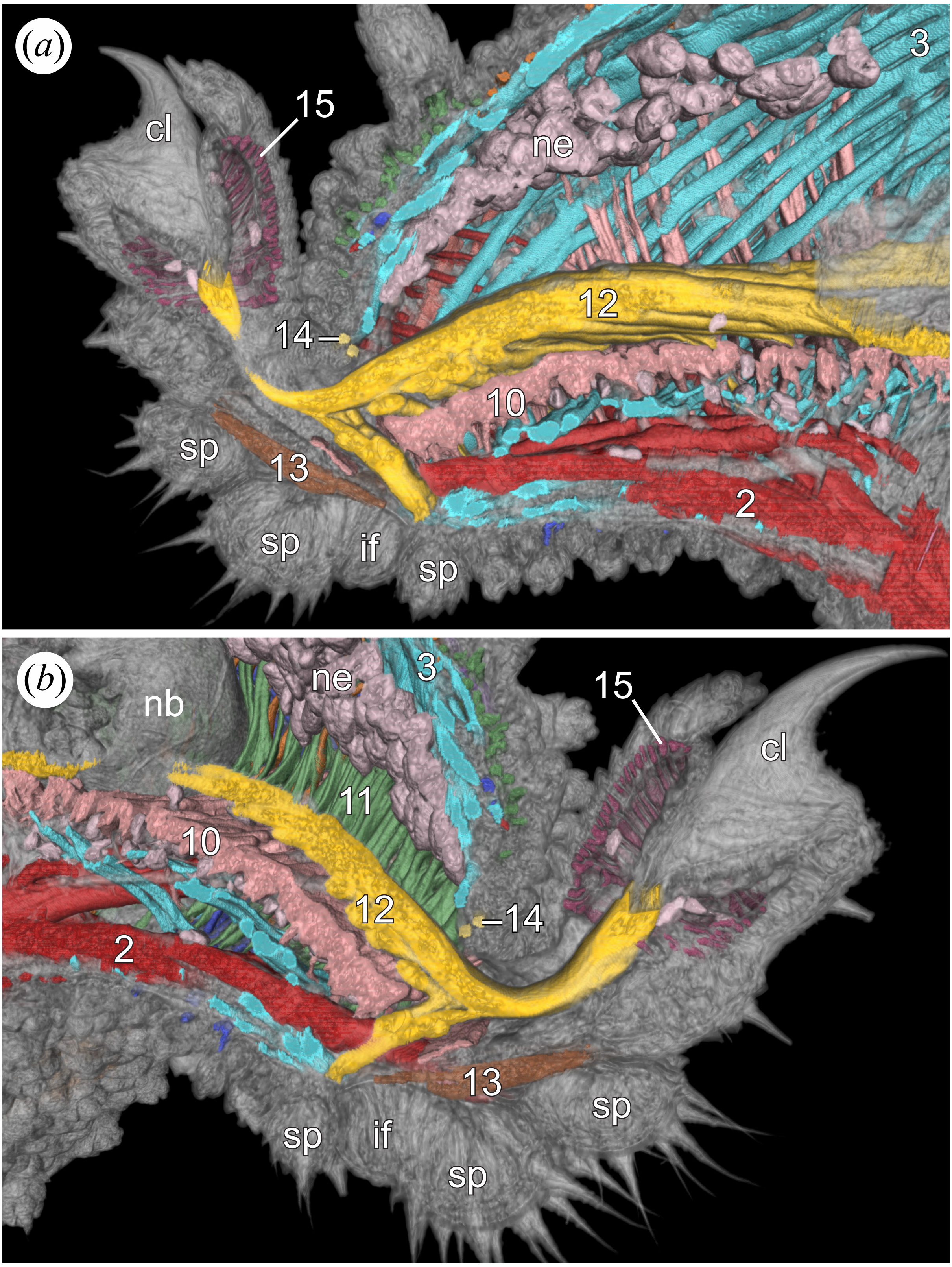

Supplementary Figure 5. Internal organisation of the lobopod in *E. rowelli*. Volume rendering based on nanoCT data from left mid-trunk leg; virtual section along the proximodistal lobopod axis. Dorsal is up in both images. (**a**) Leg in posterior view. (**b**) Leg in anterior view. Individual muscles are highlighted in different colours and numbered as in main text (summarised in Table 1). Abbreviations: cl, claw; if, spineless integumentary fold; nb, nephridial bladder; ne, nephrocytes; sp, spinous pad.
