# Supplementary material for: Functional morphology of a lobopod: case study of an onychophoran leg
Source: R Soc Open Sci. 2019 Oct 16;6(10):191200. doi: 10.1098/rsos.191200 (PMC6837196; doi:10.1098/rsos.191200)
Supplement: Figure S6 [file rsos191200supp6.pdf]

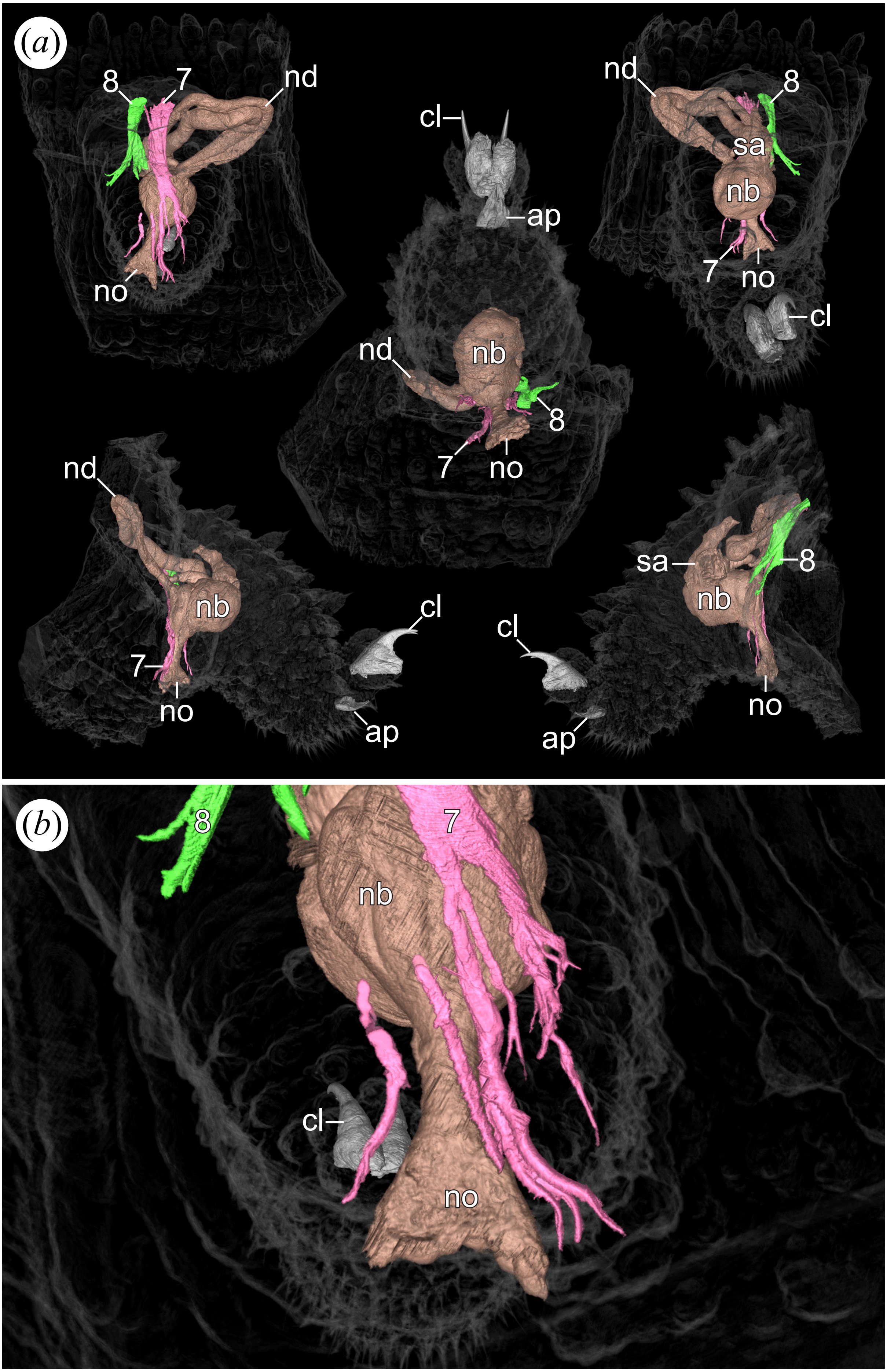


**Supplementary Figure 6. Spatial relationship between proximal leg muscles and the excretory organ in *E. rowelli*.** Volume rendering based on nanoCT data from left mid-trunk leg illustrating the anteroproximal (#7) and posteroproximal (#8) leg muscles and the nephridial system of the leg (in brown). Dorsal is up in all images except the centre image in **a** (distal is up). Body surface is semi-transparent. Note that leg muscles are not associated with the nephridial opening. (**a**) Lobopod viewed from different perspectives. (**b**) Detail of the leg viewed from trunk. Abbreviations: ap, foot apodeme; cl, claw; nb, nephridial bladder; nd, nephridial duct; no, nephridial opening; sa, sacculus.
