# Supplementary material for: Functional morphology of a lobopod: case study of an onychophoran leg
Source: R Soc Open Sci. 2019 Oct 16;6(10):191200. doi: 10.1098/rsos.191200 (PMC6837196; doi:10.1098/rsos.191200)
Supplement: Figure S7 [file rsos191200supp7.pdf]

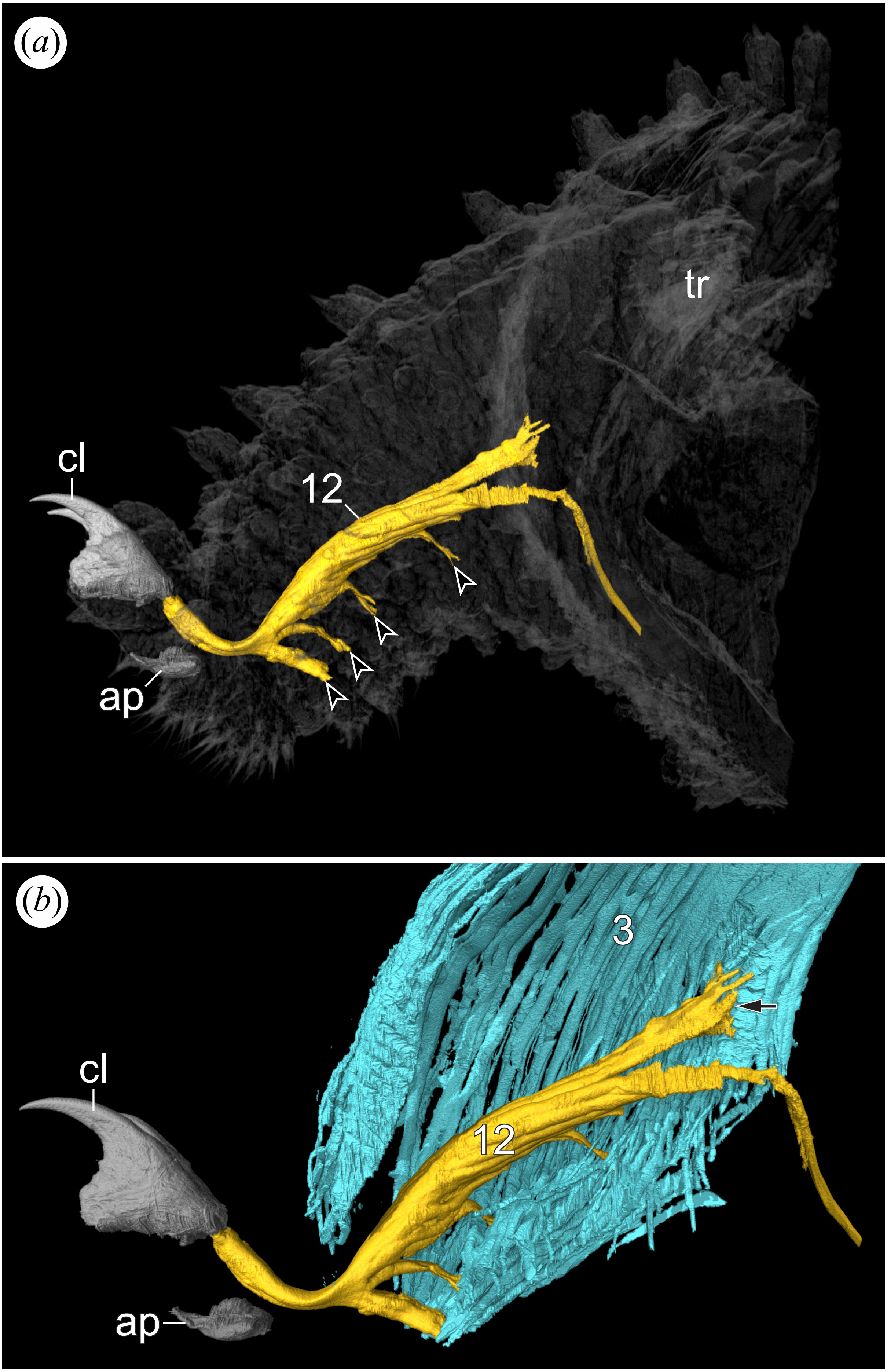

Supplementary Figure 7. Morphology of the claw retractor muscle in *E. rowelli.* Volume rendering based on nanoCT data from left mid-trunk leg. Dorsal is up in all images. Body surface is semi-transparent in **a**. (**a**) Structure and position of the claw retractor muscle (#12) inside the leg. Arrowheads point to set of muscle fibres that branch off ventrally from this muscle. (**b**) Spatial relationship between the claw retractor (#12) and the leg promotor (#3). Note that the dorsoproximal fibres of the claw retractor (arrow) fuses with those of the leg promotor. Abbreviations: ap, foot apodeme; cl, claw; tr, trunk.
