# Supplementary material for: Functional morphology of a lobopod: case study of an onychophoran leg
Source: R Soc Open Sci. 2019 Oct 16;6(10):191200. doi: 10.1098/rsos.191200 (PMC6837196; doi:10.1098/rsos.191200)
Supplement: Figure S8 [file rsos191200supp8.pdf]

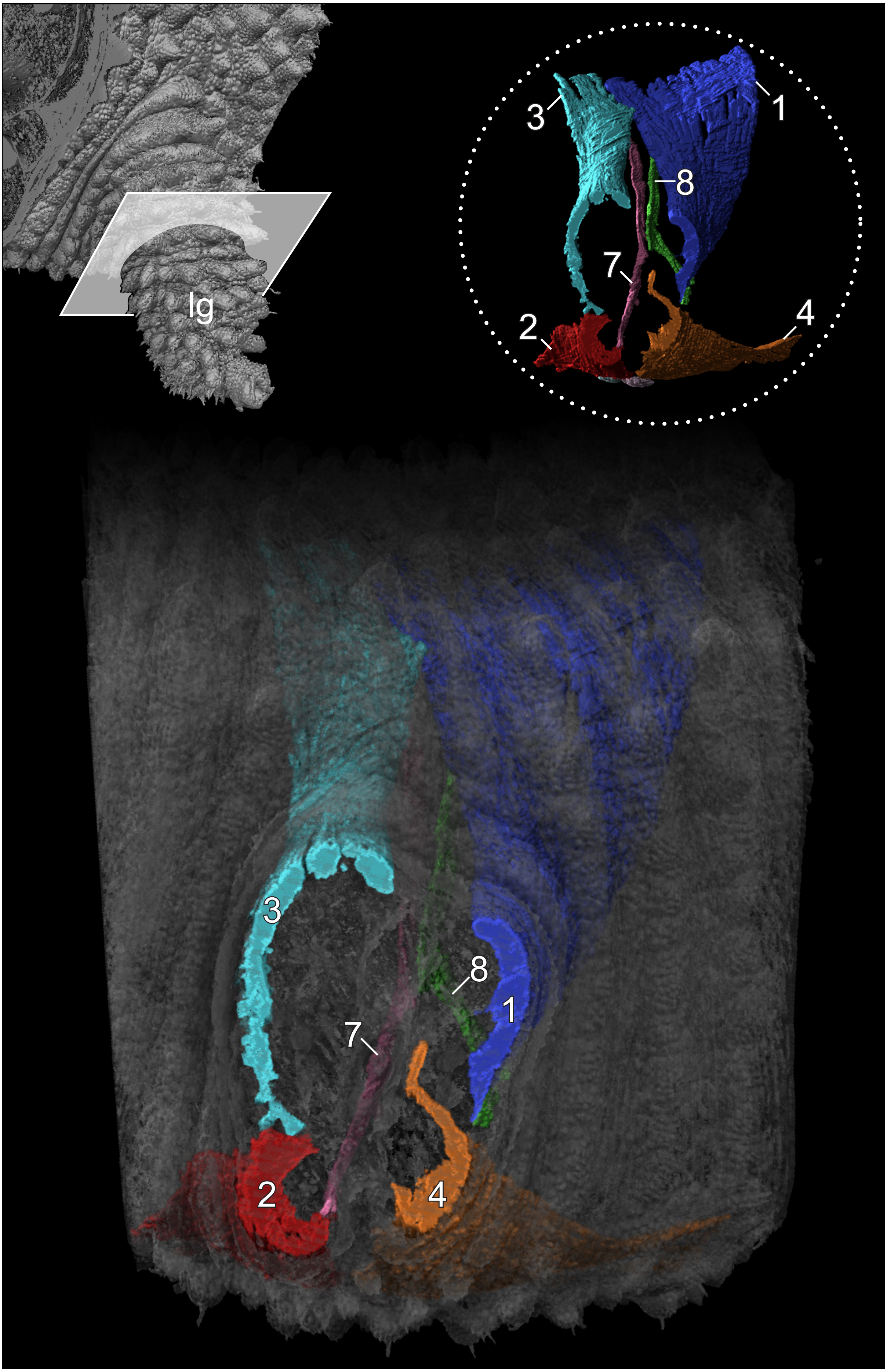

Supplementary Figure 8. Peripheral arrangement of leg muscles in *E. rowelli*. Volume rendering based on SRμCT data from left mid-trunk leg illustrating the lobopod in virtual cross section (top left image). Dorsal is up in all images. Body surface is semi-transparent (bottom image). Individual muscles are highlighted in different colours and numbered as in main text (summarised in Table 1). Abbreviation: lg, leg.
